# Trends in Medicinal Uses of Edible Wild Vertebrates in Brazil

**DOI:** 10.1155/2017/4901329

**Published:** 2017-08-15

**Authors:** Rômulo Romeu Nóbrega Alves, Tacyana Pereira Ribeiro Oliveira, Maria Franco Trindade Medeiros

**Affiliations:** ^1^Departamento de Biologia, Universidade Estadual da Paraíba, 58429-500 Campina Grande, PB, Brazil; ^2^Centro de Ciências Biológicas e Sociais Aplicadas (CCBSA), Universidade Estadual da Paraíba, 58071-160 João Pessoa, PB, Brazil; ^3^Unidade Acadêmica de Biologia e Química, Universidade Federal de Campina Grande, 58175-000 Cuité, PB, Brazil

## Abstract

The use of food medicines is a widespread practice worldwide. In Brazil, such use is often associated with wild animals, mostly focusing on vertebrate species. Here we assessed taxonomic and ecological trends in traditional uses of wild edible vertebrates in the country, through an extensive ethnobiological database analysis. Our results showed that at least 165 health conditions are reportedly treated by edible vertebrate species (*n* = 204), mostly fishes and mammals. However, reptiles stand out presenting a higher plasticity in the treatment of multiple health conditions. Considering the 20 disease categories recorded, treatment prescriptions were similar within continental (i.e., terrestrial and freshwater) and also within coastal and marine habitats, which may reflect locally related trends in occurrence and use of the medicinal fauna. The comprehension of the multiplicity and trends in the therapeutic uses of Brazilian vertebrates is of particular interest from a conservation perspective, as several threatened species were recorded.

## 1. Introduction

Wildlife represents an immeasurable source of raw materials that support health systems of different human cultures that depend on nature as a source of medicines to treat and cure illnesses [1]. Plants and animals have been used as medicinal sources since ancient times, and even today animal- and plant-based pharmacopeias continue to play an essential role in health care. Although plants and plant-derived materials make up the majority of the ingredients used in most traditional medical systems globally, whole animals, animal parts, and animal-derived products also constitute important elements of the* materia medica* [2–6].

The use of animal species as remedies, although representing an important component of traditional medicines (sometimes in association with plant species), has been much less studied than medicinal plants [1]. However, the importance of nonbotanical remedies (those of animal and mineral origin) is emerging [7], resulting in a recent boom in publications focusing on zootherapy [8–11].

Brazil is well known for its rich social/cultural diversity, as represented by more than two hundred indigenous people and a range of local communities, which in turn have contributed to the high diversity of traditional knowledge and practices which include the use of medicinal animals. Indeed, animals have been used as a source of medicine in the country and have played a significant role in healing practices as many people have used animals as medicines or alternative or supplementary treatments [12, 13].

Hence, Brazil can be considered a model to extensive zootherapeutic studies, since the use of animals and animal-derived products is widespread among the country's human cultures, as predicted by the zootherapeutic universality hypothesis [14]. Furthermore, the concomitant use of wild animals for nutritional and medicinal purposes is also diffuse in several localities in the country, thus highlighting their important role as food medicine in well-established folk medical practices [15].

Recent research has highlighted the predominant use of vertebrates as medicinal fauna in different medical systems worldwide [1]. As remarked by Perry [16], this is an expected trend, considering the frequent interactions between people and vertebrates—typically large-bodied animals, which may provide a wide range of medicinal products. This raises particular conservation concerns, as some of these taxa are overharvested for their medicinal uses and are now threatened [1].

In this article, we provide an assessment of the uses of wild edible vertebrate species in Brazilian Traditional Medicine. The study focused on the following questions: (1) which edible vertebrate taxa are mostly used in the Brazilian Traditional Medicine? (2) Do the conditions treated by medicinal resources vary with taxonomical group and/or animal's habitat?

## 2. Methods 

Data used in this research resulted from an extensive analysis of the ethnozoological database provided by the Laboratório de Etnozoologia, Universidade Estadual da Paraíba. The database comprises information from ethnozoological studies on faunal medicinal use performed in all Brazilian regions. Additional data was gathered through information available in reviews published by the laboratory researchers [17–19].

Data analysis comprised information on species of edible vertebrates used as medicines, their family classification, habitats, conservation status, and conditions to which animals were prescribed. We only considered those taxa that could be identified to species level, and the scientific nomenclature of the taxa recorded (fishes, amphibians, reptiles, and mammals) and/or habitats were in accordance with the following databases: Fishbase (Froese and Pauly, 2016; http://www.fishbase.org/), Amphibian Species of the World (http://research.amnh.org/herpetology/amphibia/index.php), The Reptile Database (http://www.reptile-database.org/), and Mammal Species of the World [20]. With regard to habitat analysis, marine and estuarine species were grouped in the same category (i.e., coastal and marine); if a marine species was also reported to freshwater environments, it habitat was categorized as costal and marine/freshwater. Moreover, continental species which could inhabit both terrestrial and aquatic systems were considered as semiaquatic species.

The conservation status of the analysed species follows the International Union for the Conservation of Nature [21], the Convention on International Trade in Endangered Species of Wild Fauna and Flora [22], and the Brazilian red lists (decrees 444 and 445, Brazilian Ministry of Environment, 2014). Health conditions considered in this research were categorized by the International Statistical Classification of Diseases and Related Health Problems (ICD-10 Version: 2016; http://apps.who.int/classifications/icd10/browse/2016/en).

### 2.1. Data Analysis

All data were verified for normal distribution (Shapiro-Wilk's test) and homogeneity of variance (Levene's test) and nonparametric tests were performed when those assumptions were not met.

A Kruskal-Wallis test (followed by Dunn's post hoc test) and an ANOVA were performed to determine whether the number of health conditions treated per species varied among vertebrate taxonomic groups or habitat types, respectively. Resemblance between health conditions treated (grouped into ICD's categories) and taxonomic groups or animals' habitat types were assessed based on Jaccard's similarity index, where resulting matrices were used to perform cluster analyses. Due to low number of species recorded (*n* = 3), amphibians were excluded from all statistical analysis regarding taxonomic groups.

## 3. Results

**Figure 1:**
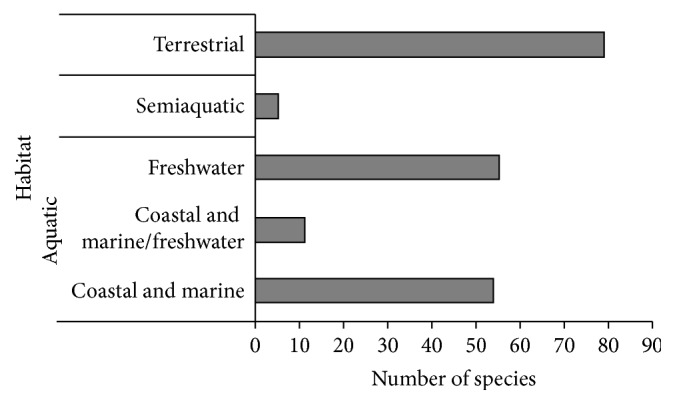
Number of edible vertebrates used in traditional medicines in Brazil, according to their habitat types.

At least 204 edible vertebrate species have been used in Brazilian Traditional Medicine (see Table 1). Fishes were the most represented group (*n* = 97 species), followed by mammals (*n* = 48), reptiles (*n* = 29), birds (*n* = 27), and amphibians (*n* = 3). Most medicinal animals are aquatic (58.9%), mostly inhabiting freshwater (27.0% of total counts) and coastal/marine (26.5% of total counts) environments (Figure 1). Terrestrial and semiaquatic vertebrates corresponded to 38.7% and 2.5% of medicinal vertebrates recorded, respectively.

**Figure 2:**
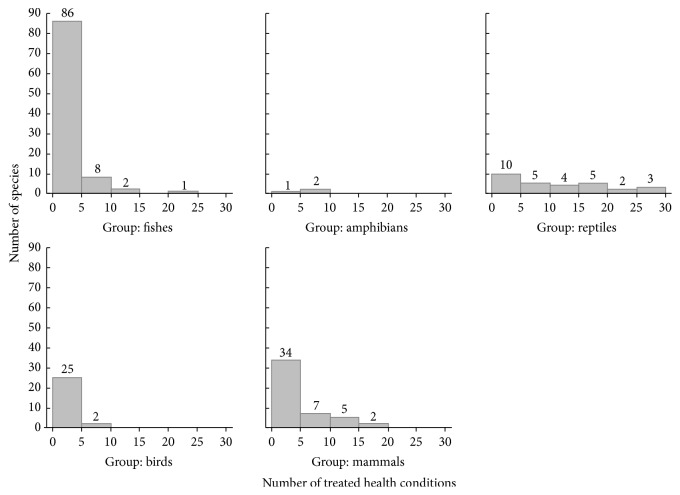
Number of health conditions treated by each taxonomic group of edible vertebrates used in traditional medicines in Brazil, according to their taxonomic group. Numbers above bars: number of animal species analysed.

Edible medicinal vertebrates were reportedly used to treat 165 health conditions/diseases (see Table 2). A single illness could be treated by various animal species (e.g., 67 animal species were used in the treatment of asthma and 60 in the treatment of rheumatism), and although most species (particularly fishes, mammals, and birds) were used to treat only one (*n* = 85; 41.7%) or up to five illnesses (*n* = 156; 76.5%), several were prescribed for treating multiple illnesses (>5 conditions; *n* = 48, 23.5%), as shown in Figure 2. Reptiles were the most versatile group, as they were mostly used in the treatment of multiple conditions, with almost half of the species (*n* = 14) being used to treat more than 10 illnesses (Figure 2). Indeed, from the 10 most expressive species in the treatment of multiple conditions (see Table 1), seven are reptiles, for instance, the “teju” and the boa snake (*Salvator teguixin* and* Boa constrictor*, resp.; *n* = 28 health conditions prescribed, each), the Neotropical rattlesnake (*Crotalus durissus*; *n* = 27 conditions), the green sea turtle (*Chelonia mydas*; *n* = 25 conditions), and the common caiman (*Caiman crocodilus*; *n* = 24 conditions). Moreover, the trahira fish (*Hoplias malabaricus*; *n* = 23 prescriptions) and the two manatee species recorded (*Trichechus inunguis* and* T. manatus*; *n* = 18 prescriptions, each) also stand out for being indicated to the treatment of multiple illnesses.

**Figure 3:**
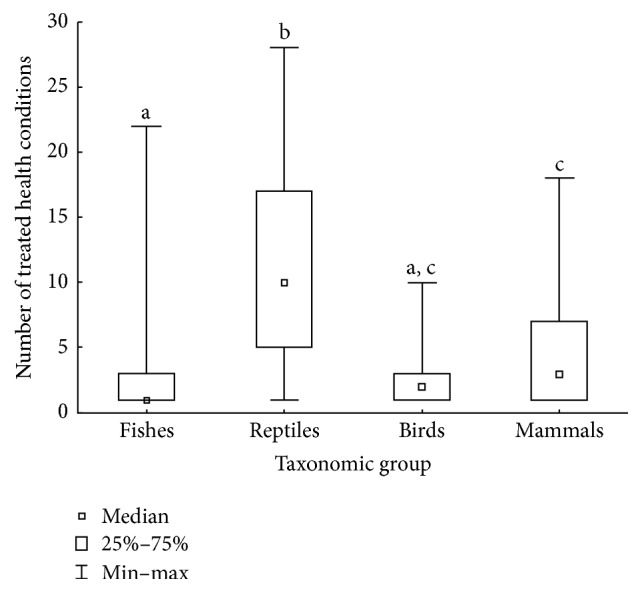
Number of health conditions treated per species, according to taxonomic group. Letters indicate the results of Dunn's post hoc test: shared letters mean no statistical difference between groups (*p* > 0.05).

Each species was prescribed to treat a mean of 4.4 ± 0.78 (mean ± confidence interval) health conditions. Reptiles contributed with the highest mean number of diseases treated per species, while birds and fishes comprised the groups with the lowest means (Kruskal-Wallis test: *H* = 53.209; *n* = 201; *p* < 0.01; Dunn's post hoc test: *p* < 0.01; Figure 3). Nonetheless, species showed similar number of prescriptions according to habitat type (*F*_4,199_ = 1.36; *p* = 0.247).

Prescriptions of edible medicinal vertebrates were generalised in 20 disease categories, according to ICD-10. From those, “symptoms, signs, and abnormal clinical and laboratory findings” were the most recorded category in terms of therapeutic quotes recorded, followed by “infectious and parasitic diseases” and “injuries, poisoning, and other consequences of external causes” (Table 2).

With regard to the number of species associated with ICD-10 categories, most animals were prescribed for treating problems associated with the “musculoskeletal system and connective tissue” and the “respiratory system” (each: *n* = 80 species; 39.2%), “injuries, poisoning, and other consequences of external causes” (67 species, 32.8%), and “symptoms, signs, and abnormal clinical and laboratory findings” (58 species, 28.4%) (Table 2).

Despite most medicinal vertebrates provide raw materials for remedies, medicinal products often have magical-religious purposes, particularly for the prevention of diseases of spiritual cause (e.g., evil eye); they were also used as amulets to prevent diseases (e.g., amulet used as a protection against snake bite). It is worth noting that many animals involved in poisoning accidents, such as stingrays and snakes, are also used in folk medicine, particularly to treat injuries caused by themselves (see Table 1).

**Figure 4:**
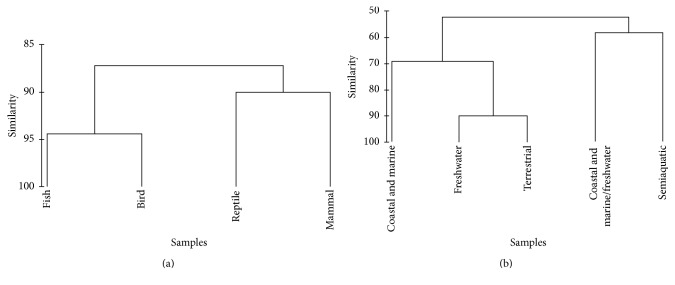
Dendogram showing taxonomic group (a) and habitat type (b) similarities with ICD-10 disease categories.

Fishes and birds appear to have most similar use according to ICD-10 categories (Jaccard index: 94.4), as well as reptiles and mammals (Jaccard index: 90.0), resulting in two distinct clusters (Figure 4(a)). When considering resemblance between the disease categories recorded and animals' habitat types, two distinct clusters were also formed (terrestrial, freshwater, coastal, and marine; costal and marine/freshwater and semiaquatic) (Figure 4(b)), thus reflecting highest similarities between continental habitats (terrestrial and freshwater; Jaccard index: 90.0).

With regard to species conservation status, 160 animals figure in at least one of the three red lists assessed (see Table 1). In the ICUN red list, 33 species (mainly fishes and mammals) are classified into threatened categories, mostly as vulnerable (VU; *n* = 27) ones. Endangered (EN) and critically endangered (CR) species comprised six fishes and reptiles, namely,* Narcine bancroftii* and* Pristis pectinata* (CR) and* Sphyrna lewini*,* S. mokarran*,* Chelonia mydas,* and* Eretmochelys imbricata* (EN). In Brazilian red lists, most threatened animals are also considered VU (*n* = 22); EN species (*n* = 9) comprise mainly fishes and mammals; and CR ones (*n* = 8) comprise mainly fishes and marine reptiles. In CITES, 58 species are listed, especially in its Appendix II (*n* = 37), mammals and reptiles being the most expressive groups.

## 4. Discussion

The high number of vertebrates used as medicine is not surprising given the important role played by wildlife as a source of medicines in different traditional medicine systems [8, 10, 23, 24]. The predominance of fishes and mammals in the Brazilian Traditional Medicine confirms our expectations, given that those groups comprise major targets in Brazil [25–28]. Although these two taxa have been primarily harvested for alimentary purposes, they generate a series of the inedible parts [such as bone, skin, tail, feather, liver, and bile (“fel”)], rattle (from rattlesnakes), spine, scale, penis, carapace, beak, teeth, head, nails, and horn that can be used in popular medicines. According to Moura and Marques [29] the use of leftover/secondary products derived from the fauna seems to be one of the most conspicuous features on the Brazilian popular zootherapy.

Zootherapeutic products, however, do not include inedible parts solely: flesh, eggs, and viscera are among some animal products used for both medicinal and alimentary purposes [1, 12, 13, 30, 31]. This corroborates the assumption that the consumption of wild vertebrates meat is often related to the purported medicinal or cultural benefits derived from the animal parts [32–35]. In a recent review study, Alves et al. [15] pointed out that at least 354 wild animal species are used in Brazilian Traditional Medicine, of which 157 are also used as food, evidencing that a close connection between eating and healing is common in Brazilian zootherapy. This is in line with several studies in ethnobiology and ethnopharmacology that have observed how difficult the clear separation between medicines and foods can be [36–38] and this situation includes plants and animals, essential items for the preparation of traditional medicine.

Whether for food or medicinal purposes, the consumption of wild animals can lead to the transmission of various human diseases [39]. Van Vliet et al. [40] highlighted that the consumption of bushmeat for either purpose may lead to human infection by several zoonotic pathogens. Armadillos, for example, are widely used in folk medicine and are a natural reservoir of etiological agents of several zoonotic diseases that affect humans, such as leprosy, trichinosis, coccidioidomycosis or Valley Fever, Chagas disease, and typhus [41]. Therefore, it is essential that traditional drug therapies are submitted to an appropriate benefit/risk analysis [39].

It was found that several medicinal vertebrates used in the Brazilian Traditional Medicine have multiple therapeutic indications. The possibility of using various remedies for the same ailment is popular because it allows adapting to the availability of the animals. The fact that some medicinal animals are being used for the same purpose suggests that different species can share similar medicinal properties and might indicate the pharmacological effectiveness of those zootherapeutic remedies [8].

Multiple medicinal uses become even more evident when considering reptiles, as this group comprises one of the most important animal resources related to the medicine history [42] and is widely used in the most important traditional pharmacopeias worldwide [35]. Indeed, the use in traditional medicines is the human practice that involves the highest diversity of reptile species in Brazil [17], some of which play important roles in traditional medicines, such as the “teju”* (Tupinambis teguixin)* and the boa snake* (Boa constrictor)*, which are one of the most used medicinal animals in Brazil [42, 43]. Curiously, there is a general aversion to consuming some reptile groups, such as snakes and lizards, in the country. Nonetheless, this fact does not impair the use of these animals as medicines, as it is mainly associated with popular beliefs known as “simpatias,” which, in most of the cases, state that “a person receiving a given treatment cannot know what that he/she is taking, otherwise the effect ceases” [18]. Hence, this fact seems to favour the high use of reptile species, despite widespread aversion to those animals.

On the other hand, despite presenting the highest diversity of medicinal species, fishes were recommended to treat a comparatively low number of health conditions. This may be related to the fact that most parts of a fish are consumed as food; thus fewer products are left to be used in medicinal practices. Similarly, when considering major hunted taxa in Brazil, that is, mammals and birds [25, 26, 44], most species are also mostly consumed as food. However, the inedible parts generate “leftovers” (e.g., skin, tail, spine, scale, teeth, nails, and horn) which are among the main products used in traditional medicine. Indeed, according to Moura and Marques [29], the zootherapeutic use of the fauna is mainly based on derived leftovers/secondary products. Those authors also emphasise that, from the ecological theory point of view, the use of leftovers could be justified as an attempt to leverage the resources obtained from ecosystems which are inappropriate for alimentary consumption due to the mechanical difficulty of ingesting these parts, such as horns, feather, and scales. Therefore, one can expect that the diversity of leftovers provided by a species may support the potential to treat multiple diseases.

Animals from continental habitats (i.e., terrestrial and freshwater) were found to treat similar disease categories; the same could be found within coastal and marine animals. This may be related to the local distribution of the diseases treated, thus leading people to use local resources in the traditional medicine of each region. For instance, in coastal areas, the occurrence of diseases classified into the category “external causes of morbidity and mortality” is very common, due to sting/poisoning accidents caused by fishes (e.g., stingrays, catfish, and toadfish), which are often treated by zootherapeutic products derived from the animals that caused the lesions [45–48].

Natural resources play an essential role in health care in traditional medical systems, as well as in bioprospecting for new drugs [49, 50], and the interest in animal-based products has raised [49, 51, 52]. Hence, despite the available information on the chemical components and actions of some of these products, studies on fauna traditional uses still are potentially very important to shed light on several aspects of their therapeutic applications [53].

The comprehension of the multiplicity and trends in therapeutic uses of several vertebrate species is of particular interest from a conservation perspective, as threatened animals, such as those recorded in this and other studies [30] could be replaced by nonthreatened species with similar properties. However, it is important to highlight that the use of animals for both food and medicinal purposes may impose higher pressure on those species under overexploitation conditions. For instance, if the animal is solely sought for medicinal purposes, it can lead the hunter/fisher to use selective capture techniques or even release nontargeted species. On the other hand, if an animal is captured for feeding reasons and is not the main target of the hunting or fishery (e.g., due to size), it can be kept by the hunter/fisher due to some medicinal property. Hence, understanding such complex interactions and trends in the use of fauna for nutritional and medicinal purposes evidences the important role that ethnobiological and ethnopharmacological studies may play in crucial discussion on the trade-offs between animal harvesting and its sustainability towards better regulation of those practices.

## 5. Conclusion

Wild edible vertebrates, particularly those inhabiting aquatic environments, are used to treat a wide range of health conditions in Brazil, with reptiles consisting of the most versatile group in multiple disease prescriptions. Moreover, a trend in prescriptions was found according to animals' habitats, as disease categories were similar within continental and within coastal and marine habitats. Several consumed species are under threat, leading to a raise in conservation concerns, particularly due to the dual function (as food and medicines) those species present.

## Figures and Tables

**Table 1:** Vertebrate species (scientific and common names, when available) used as medicine food in Brazil, with remarks to habitats, conservation status, and health conditions to which each animal is prescribed. Families and species name in alphabetical order.

Taxa	Health conditions treated	Habitat
Fishes		
Anostomidae		
*Leporinus friderici *(Bloch, 1794), threespot leporinus	Earache	Freshwater
*Schizodon knerii *(Steindachner, 1875), “piau-branco”	Leucoma, edema	Freshwater
Arapaimidae		
*Arapaima gigas* (Schinz, 1822), “arapaima,” “pirarucu,” “pirosca”	Asthma, pneumonia	Freshwater
Arhynchobatidae		
*Atlantoraja cyclophora* (Regan, 1903), eyespot skate	Haemorrhage after delivery	Marine
Ariidae		
*Aspistor luniscutis* (Valenciennes, 1840), “bagre-amarelo”	Pain relief in injuries caused by the species' sting	Marine/brackish
*Bagre bagre* (Linnaeus, 1766), coco sea catfish, “bagre-fidalgo”	Injuries caused by itself	Marine/brackish
*Genidens barbus *(Lacepède, 1803), white sea catfish, “bagre-do-mangue”	Pain relief in injuries caused by the species' sting	Marine/brackish
*Genidens genidens *(Cuvier, 1829), sea catfish, “bagre”	Injuries caused by itself	Marine/brackish
Aspredinidae		
*Aspredinichthys tibicen *(Valenciennes, 1840), tenbarbed banjo, “viola”	Asthma	Freshwater/brackish
*Aspredo aspredo *(Linnaeus, 1758), banjo catfish, “banjo,” “viola”	Asthma	Freshwater/brackish
Auchenipteridae		
*Trachelyopterus galeatus* (Linnaeus, 1766),“cumbá”	Umbilical hernia, asthma, sexual impotence	Freshwater
Balistidae		
*Balistes capriscus *Gmelin, 1789, grey triggerfish, “peixe-porco”	Bronchitis	Marine/reef
*Balistes vetula *Linnaeus, 1758, queen triggerfish, “cangulo,” “capado,” “peroá”	Stroke, asthma, thrombosis, earache, pain relief in injuries caused by the species' sting, haemorrhage, ascites, schistosomiasis, appendicitis, menstrual cramps, gastritis	Marine/reef
Batrachoididae		
*Thalassophryne nattereri* Steindachner, 1876, venomous toadfish, “niquim”	Pain relief caused in injuries by the species' sting	Marine/brackish
Bryconidae		
*Brycon nattereri* Günther, 1864, “pirapitinga,” “matrinchã”	Flu	Freshwater
Callichthyidae		
*Callichthys callichthys *(Linnaeus, 1758), “cascarudo,” “caboge”	Asthma, umbilical hernia	Freshwater
Lamnidae		
*Carcharodon carcharias* (Linnaeus, 1758), great white shark	Osteoporosis	Marine/brackish
*Isurus oxyrinchus* Rafinesque, 1810, shortfin mako	Osteoporosis	Marine
Carcharhinidae		
*Carcharhinus falciformis *(Müller & Henle, 1839), silky shark	Osteoporosis	Marine/reef
*Carcharhinus leucas *(Müller & Henle, 1839), bull shark, “tubarão-cabeça-chata”	Osteoporosis	Marine/brackish/reef/freshwater
*Carcharhinus limbatus* (Müller & Henle, 1839), blacktip shark, “sucuri preto”	Osteoporosis	Marine/brackish/reef
*Carcharhinus obscurus *(Lesueur, 1818), dusky shark	Osteoporosis	Marine/brackish/reef
*Carcharhinus porosus *(Ranzani, 1839), smalltail shark, “junteiro,” “cação-galha-preta”	Asthma, rheumatism, wounds, inflammations, osteoporosis, anaemia	Marine/brackish
*Galeocerdo cuvier* (Péron & Lesueur, 1822), tiger shark, “jaguara”	Osteoporosis	Marine/brackish/reef
*Negaprion brevirostris* (Poey, 1868), lemon shark	Osteoporosis	Marine/brackish/reef
*Rhizoprionodon lalandii* (Müller & Henle, 1839), Brazilian sharpnose shark, “cação”	Rheumatism, osteoporosis	Marine
*Rhizoprionodon porosus* (Poey, 1861), Caribbean sharpnose shark, “cação”	Rheumatism, osteoporosis	Marine/brackish/reef/freshwater
Sphyrnidae		
*Sphyrna lewini *(Griffith & Smith, 1834), scalloped hammerhead, “peixe-martelo,” “cação-panã,” “cação-chapéu”	Asthma, wounds, rheumatism, inflammation	Marine/brackish/reef
*Sphyrna mokarran* (Rüppell, 1837), great hammerhead	Osteoporosis	Marine/brackish/reef
*Sphyrna zygaena* (Linnaeus, 1758), smooth hammerhead	Osteoporosis	Marine/brackish
Squalidae		
*Squalus cubensis *Howell Rivero, 1936, Cuban dogfish	Osteoporosis	Marine
Rhinobatidae		
*Rhinobatos percellens *(Walbaum, 1792), Chola guitarfish	Osteoporosis	Marine
Centropomidae		
*Centropomus parallelus* Poey, 1860, fat snook	Nephritis	Marine/brackish/freshwater
*Centropomus undecimalis *(Bloch, 1792), common snook, “rubalão”	Edema in the legs	Marine/brackish/reef/freshwater
Characidae		
*Astyanax bimaculatus *(Linnaeus, 1758), twospot astyanax, “piaba-mirim,” “machadinha,” “piaba chata”	Alcoholism, leishmaniosis, skin burns, wounds, rheumatism	Freshwater
Clupeidae		
*Harengula jaguana* (Poey, 1865), scaled herring, “sardinha”	Osteoporosis	Marine/brackish/reef
*Opisthonema oglinum* (Lesueur, 1818), Atlantic thread herring, “sardinha”	Alcoholism, osteoporosis	Marine/reef
Cynodontidae		
*Hydrolycus scomberoides* (Cuvier, 1816), Payara, “cachorra”	Earache	Freshwater
Dasyatidae		
*Dasyatis guttata *(Bloch & Schneider, 1801), longnose stingray, “raia branca”	Asthma, pain relief in injuries caused by the species' sting, burns	Marine
*Dasyatis marianae *Gomes, Rosa & Gadig, 2000, Brazilian large-eyed stingray, “raia mariquita,” “raia de fogo”	Asthma, pain relief in injuries caused by the species' sting, burns	Marine/reef
Diodontidae		
*Chilomycterus antillarum *Jordan & Rutter, 1897, web burrfish	Wounds, lump	Marine/reef
*Chilomycterus spinosus spinosus *(Linnaeus, 1758)	Wounds, lump	Marine/brackish
Doradidae		
*Franciscodoras marmoratus* (Lütken, 1874), “Urutu”	Injuries caused by itself	Freshwater
*Lithodoras dorsalis* (Valenciennes, 1840), Rock-bacu	Swelling	Freshwater
*Megalodoras uranoscopus* (Eigenmann & Eigenmann, 1888), “cuiu-cuiu”	Rheumatism	Freshwater
*Oxydoras niger* (Valenciennes, 1821), ripsaw catfish, “cuiu-cuiu”	Rheumatism	Freshwater
*Platydoras costatus* (Linnaeus, 1758), Raphael catfish, “cuiu-cuiu”	Rheumatism	Freshwater
*Pterodoras granulosus* (Valenciennes, 1821), granulated catfish, “cuiu-cuiu”	Rheumatism	Freshwater
Echeneidae		
*Echeneis naucrates* Linnaeus, 1758, live sharksucker, “rêmora,” “pegador”	Asthma, bronchitis	Marine/brackish/reef
*Remora remora* (Linnaeus, 1758), shark sucker, “rêmora”	Osteoporosis	Marine/reef
Erythrinidae		
*Erythrinus erythrinus* (Bloch & Schneider, 1801), “Matrôe”	Asthma	Freshwater
*Hoplias malabaricus *(Bloch, 1794),trahira, “traíra”	Ophthalmological problems, rheumatism, cataracts, wounds, snake bite, conjunctivitis, stroke, thrombosis, asthma, toothache, fever, earache, diarrhoea, deafness, boils, bleeding, alcoholism, tetanus, sore throat, itching, sprains, leucoma	Freshwater
*Hoplias aimara *(Valenciennes, 1847)	Earache	Freshwater
Cichlidae		
*Cichla melaniae * Kullander & Ferreira, 2006	Pain relief in injuries caused by the ray sting	Freshwater
Gadidae		
*Gadus morhua* Linnaeus, 1758, Atlantic cod, “bacalhau”	Boils	Marine/brackish
Ginglymostomatidae		
*Ginglymostoma cirratum *(Bonnaterre, 1788),nurse shark, “cação-lixa”	Rheumatism, osteoporosis	Marine/brackish/reef
Gymnotidae		
*Electrophorus electricus *(Linnaeus, 1766), electric eel, “poraquê”	Sprains, bruises, insect bites, snake bite, asthma, flu, pain in general, muscle strain, rheumatism, osteoporosis, deafness, pneumonia, itching	Freshwater
Heptapteridae		
*Pimelodella brasiliensis* (Steindachner, 1876), “mandim”	Injuries caused by that fish species	Freshwater
Holocentridae		
*Holocentrus adscensionis* (Osbeck, 1765), squirrelfish, “jaguaricá”	Wounds	Marine/reef
Megalopidae		
*Megalops atlanticus *Valenciennes, 1847, tarpon, “camurupim,” “cangurupim”	Stroke, headache, asthma, shortness of breath, thrombosis, chest pain, injuries caused by bang	Marine/brackish/reef/freshwater
Monacanthidae		
*Cantherhines macrocerus *(Hollard, 1853), American whitespotted filefish	Asthma	Marine/reef
*Monacanthus ciliatus *(Mitchill, 1818), fringed filefish	Asthma	Marine/reef
Muraenidae		
*Gymnothorax funebris* Ranzani, 1839, green moray, “moréia verde”	Bleeding (wounds)	Marine/reef
*Gymnothorax moringa *(Cuvier, 1829), spotted moray, “moréia pintada”	Bleeding (wounds)	Marine/reef
*Gymnothorax vicinus *(Castelnau, 1855), purplemouth moray, “moréia”	Bleeding (wounds)	Marine/reef
Myliobatidae		
*Aetobatus narinari *(Euphrasen, 1790), spotted eagle ray, “raia-chita”	Asthma, pain relief in injuries caused by the species' sting, burns, haemorrhage	Marine/reef
Narcinidae		
*Narcine bancroftii *(Griffith & Smith, 1834), lesser electric ray	Pain	Marine
*Narcine brasiliensis *(Olfers, 1831), Brazilian electric ray, “raia elétrica”	Toothache	Marine/reef
Pimelodidae		
*Phractocephalus hemioliopterus *(Bloch & Schneider, 1801), redtail catfish, “Pirarara”	Asthma, wounds, hernia, burns in the skin, rheumatism, flu, cough	Freshwater
*Pseudoplatystoma corruscans* (Spix & Agassiz, 1829), spotted sorubim, “surubim”	Flu, removal of wrath	Freshwater
*Pseudoplatystoma fasciatum* (Lunnaeus, 1776), barred sorubim, “surubim”	Cold	Freshwater
*Sorubimichthys planiceps *(Spix & Agassiz, 1829), firewood catfish, “surubim chicote”	Leishmaniosis, tuberculosis	Freshwater
*Zungaro zungaro *(Humboldt, 1821), gilded catfish, black manguruyu, “jaú”	Asthma, toothache, earache, wounds, athlete's foot, burns in the skin, rheumatism, flu	Freshwater
Potamotrygonidae		
*Paratrygon aiereba* (Müller & Henle, 1841), discus ray,“arraia”	Asthma, hernia, flu, pneumonia, cough, earache, burns	Freshwater
*Plesiotrygon iwamae *Rosa, Castello & Thorson, 1987, long-tailed river stingray	Pain relief in injuries caused by the species' sting, wounds, cracks in the sole of the feet	Freshwater
*Potamotrygon hystrix *(Müller & Henle, 1834),porcupine river stingray, “arraia”	Asthma, hernia, flu, pneumonia, cough, earache, burns	Freshwater
*Potamotrygon motoro* (Müller & Henle, 1841), South American freshwater stingray, ocellate river stingray, “arraia”	Asthma, hernia, flu, pneumonia, cough, earache, burns	Freshwater
*Potamotrygon orbignyi *(Castelnau, 1855), smooth back river stingray, “arraia”	Pain relief in injuries caused by that species' sting	Freshwater
Pristidae		
*Pristis pectinata *Latham, 1794, smalltooth sawfish, “espadarte,” “peixe-serra”	Asthma, rheumatism, arthritis	Marine/brackish/freshwater
*Pristis perotteti *Müller & Henle, 1841, largetooth sawfish, “espadarte”	Asthma, rheumatism, arthritis	Marine/brackish/freshwater
Prochilodontidae		
*Prochilodus argenteus* Spix & Agassiz, 1829, “curimatá-pacú," “curimatá”	To avoid swelling of the breast feeding, mycosis	Freshwater
*Prochilodus nigricans* Spix & Agassiz, 1829, black prochilodus, “curimatã”	Chilblain, skin burns, wounds, rheumatism, eye pains	Freshwater
Serrasalmidae		
*Colossoma macropomum* (Cuvier, 1818), cachama, “tambaqui”	Paralysis of arms and legs	Freshwater
*Mylossoma duriventre *(Cuvier, 1818), pacupeba, “pacu-manteiga”	Venereal disease	Freshwater
*Serrasalmus brandtii* Lütken, 1875, white piranha, “pirambeba”	Inflammations, sexual impotence	Freshwater
Sciaenidae		
*Cynoscion acoupa* (Lacepède, 1801), acoupa weakfish, “pescada amarela”	Renal failure	Marine/brackish/freshwater
*Cynoscion leiarchus* (Cuvier, 1830), smooth weakfish, “pescada branca”	Renal failure	Marine/brackish
*Micropogonias furnieri *(Desmarest, 1823), whitemouth croaker, “corvina”	Pain relief in injuries caused by the species' sting, cough, asthma, bronchitis	Marine/brackish
*Pachyurus francisci* (Cuvier, 1830), San Francisco croaker, “cruvina-de-bico”	Asthma, urinary incontinence, backache	Freshwater
*Plagioscion squamosissimus *(Heckel, 1840), South American silver croaker, “curvina”	Urinary disorders, haemorrhage, snake bites	Freshwater
*Plagioscion surinamensis* (Bleeker, 1873), Bashaw,“pacora,” “curvina”	Urinary disorders, haemorrhage, snake bites	Freshwater
Sparidae		
*Calamus penna *(Valenciennes, 1830), sheepshead porgy, “peixe-pena”	Asthma	Marine/reef
Synbranchidae		
*Synbranchus marmoratus *Bloch, 1795, marbled swamp eel,“muçum”	Bronchitis	Freshwater/brackish
Tetraodontidae		
*Colomesus psittacus* (Bloch & Schneider, 1801), banded puffer, “baiacu”	Breast cancer, backache, warts	Marine/brackish/freshwater
*Sphoeroides testudineus *(Linnaeus, 1758), checkered puffer, “baiacu”	Rheumatism	Marine/brackish/reef
Trichiuridae		
*Trichiurus lepturus* Linnaeus, 1758, largehead hairtail	Asthma	Marine/brackish
Urotrygonidae		
*Urotrygon microphthalmum *Delsman, 1941, smalleyed round stingray, “raia”	Asthma, pain relief in injuries caused by the species' sting, burns	Marine
Amphibians		
Bufonidae		
*Rhinella jimi* (Stevaux 2002)	Urinary incontinence, dental caries, cancer, wounds, boils, erysipelas acne, inducing abortion	Semiaquatic
Leptodactylidae		
*Leptodactylus *cf.* labyrinthicus* (Spix, 1824), South American pepper frog, “jia-de-peito,” “rã- pimenta”	Earache, rheumatism, joint pain, cancer, sore throat	Semiaquatic
*Leptodactylus vastus *Lutz, 1930, South American pepper frog, ra-pimenta	Sore throat, cough, asthma, arthritis, backache, tonsillitis, hoarseness	Semiaquatic
Reptiles		
Iguanidae		
*Iguana iguana *(Linnaeus, 1758), Common iguana, “camaleão”	Earache, erysipelas, asthma, rheumatism, edema, abscesses, joint pain, wounds, acne, athlete's foot, sore throat, swelling, burn, tumour, sucking a splinter out of skin or flesh, boil, injuries caused by the spines of the “arraia” and others fishes, inflammation, hernia	Terrestrial
Teiidae		
*Tupinambis merianae* (Duméril & Bibron, 1839), Lizard, “tegu,” “tejuaçú”	Earache, deafness, rheumatism, erysipelas, skin thorns and wounds, respiratory diseases, sore throat, snake bite, asthma, tumour, swelling, infection, bronchitis	Terrestrial
*Tupinambis teguixin *(Linnaeus 1758), Lizard, “tegu,” “tejuaçú”	Sexual impotence, rheumatism, erysipelas, dermatitis, snake bites, asthma, tetanus, earache, thrombosis, wounds, infection of nail, swelling, herpes zoster, irritation when milk teeth are erupting, jaundice, inflammation, tumour, sore throat, infection, bronchitis, injuries caused by the spines of the “arraia,” pain relief in injuries caused by snake bites, toothache, sucking a splinter out of skin or flesh, headache, cough, stroke, coarse throat	Terrestrial
Boidae		
*Boa constrictor *(Linnaeus, 1758), Boa, “jibóia”	Rheumatism, lung disease, thrombosis, boils, tuberculosis, stomach ache, edema, snake bite, cancer, ache, swelling, helping to prevent abortion, pain in the body, inflammation, athlete's foot, calluses, tumours, cracks in the sole of the feet, goitre, sore throat, arthrosis, insect sting, dog bite, erysipelas, thrombosis, asthma, neck strain, strain muscle	Terrestrial
*Corallus hortolanus* (Linnaeus, 1758), snake	Assisting in removing spines or other sharp structures from the skin, rheumatism	Terrestrial
*Eunectes murinus *(Linnaeus, 1758), anaconda, “sucurujú,” “sucuri”	Wounds, skin problems, bruises, sprains, arthrosis, rheumatism, boils, sexual impotence, headache, sore throat, thrombosis, swelling, tumour, asthma, muscle strain, numbness, syphilis, reducing pain, luxation	Semiaquatic
*Epicrates cenchria *(Linnaeus, 1758), Brazilian rainbow boa, “salamanta”	Rheumatism, pain in articulations, injuries caused by itself, sore throat, earache	Terrestrial
Crotalidae		
*Crotalus durissus *(Linnaeus, 1758), Neotropical rattlesnake, “cascavel”	Asthma, snake bite, thrombosis, wounds, luxation, rheumatism, pain in the legs, erysipelas, deafness, epilepsy, skin diseases, tuberculosis, hanseniasis, backache, tumour, boil, headache, earache, osteoporosis, sore throat, toothache, pain relief in injuries caused by sting of insects and snake bite, irritation when milk teeth are erupting, tonsillitis, impotence, fatigue	
Chelidae		
*Phrynops geoffroanus *(Schweigger, 1812), Geoffroy's side-necked turtle, “cágado”	Asthma, sore throat, swelling, earache, rheumatism, arthrosis, healing of umbilical cord of newborn baby, mumps	Freshwater
*Phrynops tuberosus *(Peters, 1870)	Diphtheria, headache, earache, pain in the breast, wounds, furuncle, gastritis, swelling, haemorrhoids, sore throat, backache, eye problems, sucking a splinter out of skin or flesh, rheumatism, deafness	Freshwater
*Mesoclemmys tuberculata* (Luederwaldt, 1926), tuberculate toadhead turtle, “cágado,” “cágado-d'água”	Rheumatism, discharge, thrombosis, bronchitis, diarrhoea, haemorrhage, asthma, sore throat, hoarseness, muscle aches	Freshwater
Cheloniidae		
*Caretta caretta *(Linnaeus, 1758), loggerhead turtle, “tartaruga cabeçuda”	Injuries caused by bang, toothache, diabetes, headache, backache, wounds, cough, bronchitis, asthma, thrombosis, rheumatism, stroke, hoarseness, flu, backache, earache, sore throat, swelling	Marine
*Chelonia mydas *(Linnaeus, 1758), green sea turtle, “tartaruga verde,” “aruanã”	Injuries caused by bang, toothache, diabetes, headache, backache, wounds, cough, bronchitis, asthma, flu, thrombosis, rheumatism, toothache, stroke, hoarseness, earache, sore throat, swelling, whooping cough, arthritis, erysipelas, boil, wounds, arthrosis, inflammation	Marine
*Eretmochelys imbricata *(Linnaeus, 1766), Atlantic hawksbill, “tartaruga de pente”	Injuries caused by bang, toothache, diabetes, headache, backache, wounds, cough, bronchitis, asthma, thrombosis, stroke, hoarseness, flu, rheumatism, earache, sore throat, swelling	Marine
*Lepidochelys olivacea *(Eschscholtz, 1829), olive ridley	Injuries caused by bang, toothache, diabetes, headache, backache, wounds, cough, flu, bronchitis, asthma, thrombosis, rheumatism, stroke, hoarseness	Marine
Dermochelyidae		
*Dermochelys coriacea *(Vandelli, 1761), leatherback turtle, “tartaruga de couro”	Rheumatism, earache, sore throat, swelling	Marine
Geoemydidae		
*Rhinoclemmys punctularia* (Daudin, 1802), spot-legged turtle	Wounds, tumour, erysipelas, earache, rheumatism	Semiaquatic
Podocnemididae		
*Podocnemis expansa* (Schweiger, 1812), Amazon river turtle, “tartaruga da amazônia”	Inflammation, acne, tumour, boil, rheumatism, pterygium, skin spots, backache, earache, arthrosis, arthritis, swelling, wrinkle	Freshwater
*Podocnemis unifilis* (Troschel, 1848), yellow-spotted river turtle, “tracajá”	Wounds, tumour, erysipelas, earache, rheumatism	Freshwater
*Podocnemis sextuberculata* Cornalia, 1849, six-tubercled Amazon River turtle	Blackhead, acne	Freshwater
*Peltocephalus dumeriliana* Schweigger 1812, “Cabeçuda”	Blackhead, acne	Freshwater
Testudinidae		
*Chelonoidis carbonaria *(Spix, 1824), red-footed tortoise, “jabuti”	Catarrh, erysipelas, bronchitis, stopping the sensation of getting thirsty, asthma	Terrestrial
*Chelonoidis denticulata *(Linnaeus, 1766), yellow-footed tortoise, “jabuti”	Sore throat, rheumatism, hernia, wounds, leishmaniosis, varicocele, earache	Terrestrial
Kinosternidae		
*Kinosternon acutum *(Linnaeus 1766), Tabasco Mud Turtle	Muscle aches	Freshwater
Alligatoridae		
*Caiman crocodilus* (Linnaeus, 1758), common cayman, “jacaré tinga”	Asthma, stroke, bronchitis, backache, earache, rheumatism, thrombosis, sexual impotence, snake bites (antidote), evil eye, irritation when milk teeth are erupting, discharge, swelling, scratch, athlete's foot, ophthalmological problems, asthma, sore throat, amulet used as a protection against snake bite, rheumatism, hernia, prostate problems, infection, thrombosis	Freshwater
*Caiman latirostris *(Daudin, 1801), broad-snouted caiman, “jacaré-do-papo-amarelo”	Asthma, sore throat, amulet used as a protection against snake bite, rheumatism, irritation when milk teeth are erupting, hernia, prostate problems	Freshwater
*Melanosuchus niger *(Spix, 1825), black caiman, “jacare açú”	Thrombosis, infection, swelling, asthma, amulet used as a protection against snake bite, injuries caused by spines of the “arraia,” pain relief in injuries caused by snake bites	Freshwater
*Paleosuchus palpebrosus* (Cuvier, 1807), dwarf caiman, “jacaré coroa,” “jacaré,” “jacaré-preto,” “crocodilo”	Snake bite, asthma, stroke, rheumatism, thrombosis, backache, sexual impotence, edema, mycosis, evil eye, irritation when milk teeth are erupting, snake bite (antidote), discharge, sore throat, amulet used as a protection against snake bite, hernia, prostate problems	Freshwater
*Paleosuchus trigonatus* (Schneider, 1801), smooth-fronted caiman, “Jacaré coroa”	Rheumatism	Freshwater
Birds		
Anatidae		
*Sarkidiornis sylvicola* H. Ihering & R. Ihering, 1907, American Comb Duck, “putrião”	Bleeding (wounds)	Terrestrial
Anhimidae		
*Anhima cornuta* (Linnaeus, 1766), horned screamer, “anuhma”	Intoxication from poisonous animals	Terrestrial
Ardeidae		
*Ardea cocoi *(Linnaeus, 1766), white-necked Heron	Swelling, inflammation, injuries caused by the spines of the “arraia” and others fishes, asthma, boil, tumour, inflammation, rheumatism, earache	Terrestrial
Caprimulgidae		
*Nyctidromus albicollis* (Gmelin, 1789), pauraque, “bacurau”	Amulets, snake bite	Terrestrial
Cracidae		
*Penelope jacucaca* (Spix, 1825), white-browed guan, “jacu”	Insomnia, epilepsy	Terrestrial
*Pauxi tuberosa *(Spix, 1825), razor-billed Curassow	Bleeding, snakebite, indigestion, stroke, lack of appetite in children, pneumonia	Terrestrial
Ciconiidae		
*Ciconia maguari* (Gmelin, 1789), maguari stork	Injuries caused by the spines of the “arraia” and others fishes, thrombosis	Terrestrial
Columbidae		
*Columbina minuta *(Linnaeus, 1766), plain-breasted ground dove	Lack of appetite, nausea during pregnancy	Terrestrial
*Columbina picui *(Temminck, 1813), Picui Dove	Lack of appetite, nausea during pregnancy, deafness	Terrestrial
*Columbina talpacoti *(Temminck, 1810), Ruddy Ground Dove, “rolinha-caldo-de-feijão”	Lack of appetite, nausea during pregnancy, deafness	Terrestrial
*Leptotila rufaxilla *(Richard & Bernard, 1792), Grey-Fronted Dove, “juriti”	Lack of appetite, nausea during pregnancy, deafness, stye, thrombosis	Terrestrial
Corvidae		
*Cyanocorax cyanopogon* (Wied, 1821), white-naped jay, “can-can”	Asthma	Terrestrial
Cuculidae		
*Crotophaga ani* Linnaeus, 1758, smooth-billed ani	Bronchitis, thrombosis, asthma, whooping cough	Terrestrial
*Guira guira *(Gmelin, 1788), guira cuckoo, “anum branco”	Asthma	Terrestrial
Charadriidae		
*Vanellus chilensis *(Molina, 1782), southern lapwing, “quero-quero”	Helping to stay awake	Terrestrial
Emberizidae		
*Coereba flaveola* (Linnaeus, 1758), bananaquit, “caga-sebo”	Thrombosis	Terrestrial
Furnariidae		
*Furnarius rufus* (Gmelin, 1788), rufous hornero, “maria-barreira”	Mumps	Terrestrial
*Podicipedidae*		
*Tachybaptus dominicus *(Linnaeus, 1766), Least Grebe, “mergulhão-pequeno,” “mergulhão,” “mergulhão-preto”	Eye problems	Semiaquatic
Rallidae		
*Aramides cajanea* (Statius Müller, 1776), grey-necked wood-rail, “saracura”	Evil eye	Terrestrial
Rheidae		
*Rhea americana* (Linnaeus, 1758), greater rhea, “ema”	General aches, rheumatism, thrombosis, strokes	Terrestrial
Tinamidae		
*Crypturellus noctivagus* (Wied, 1820), yellow-legged tinamou,“zabele”	Thrombosis, stroke	Terrestrial
*Crypturellus tataupa * (Temminck, 1815), Tataupa Tinamou	Assisting children who take longer than usual to start walking	Terrestrial
*Crypturellus parvirostris * (Wagler, 1827), small-billed Tinamou	Assisting children who take longer than usual to start walking	Terrestrial
*Nothura boraquira* (Spix, 1825), white-bellied nothura, “codorna”	Thrombosis, stroke, earache	Terrestrial
*Nothura maculosa *(Temminck, 1815), Spotted Nothura, “lambú espanta-boiada,” “lambú-de-capoeira”	Snake bite	Terrestrial
*Tinamus tao *Temminck, 1815, Grey Tinamou	Snake bite	Terrestrial
*Rhynchotus rufescens *(Temminck, 1815), red-winged tinamou,“perdiz”	Thrombosis, snake bites (antidote)	Terrestrial
Mammals		
Agoutidae		
*Cuniculus paca *(Linnaeus, 1766), spotted paca, “paca”	Wound in the breast caused by suckling, ophthalmological problems, stomach disorders, pterygium, sucking a splinter out of skin or flesh, injuries caused by the spines of “arraia,” control of cholesterol level	Terrestrial
Bovidae		
*Bubalus bubalis* (Linnaeus, 1758), water buffalo (feral), “búfalo”	Rheumatism, osteoporosis, thrombosis	Terrestrial
Bradypodidae		
*Bradypus variegatus* Shinz, 1825, brown-throated three-toed sloth, “Preguiça pequena”	Thrombosis	Terrestrial
*Bradypus tridactylus* Linnaeus, 1758, pale-throated three-toed sloth, “Preguiça”	Thrombosis, insects bite, scorpions bite	Terrestrial
Canidae		
*Cerdocyon thous *(Linnaeus, 1766), crab-eating fox, “raposa”	Rheumatism, flu, haemorrhoids, disorders after parturition (to accelerate recovery after parturition)	Terrestrial
*Chrysocyon brachyurus* (Illiger, 1815), maned wolf, “lobo-guará”	Epilepsy	Terrestrial
*Dusicyon thous* Linnaeus, 1766, crab-eating fox, “raposa”	Alcoholism, thrombosis, rheumatism, ophthalmological problems, diabetes, urinary infection	Terrestrial
Caviidae		
*Cavia aperea* Erxleben, 1777, Brazilian Guinea Pig, “Preá”	Inflammation	Terrestrial
*Galea spixii *(Wagler, 1831), Spix's Yellow-Toothed Cavy	Inflammation	Terrestrial
*Kerodon rupestris* (Wied-Neuwied, 1820), Rock Cavy, “Mocó”	Constipation	Terrestrial
Cebidae		
*Alouatta belzebul* (Linnaeus, 1766), red-handed howler monkey, “guariba,” “macaco”	Whooping cough, sore throat, asthma	Terrestrial
*Alouattanigerrima* Lönnberg, 1941, Black Howler Monkey	Whooping cough, inflammation	Terrestrial
*Alouatta macconnelli *(Linnaeus, 1766), red howler monkey, “guariba vermelho”	Whooping cough, inflammation, accelerating parturition	Terrestrial
*Sapajus apella *(Linnaeus, 1758), brow capuchin, “capuchin,” “macaco,” “macaco-prego”	Insect sting	Terrestrial
Cervidae		
*Blastocerus dichotomus* (Illiger, 1815), marsh deer, “cervo-do-pantanal”	Diarrhoea, vomit	Terrestrial
*Mazama americana* (Erxleben, 1777), red brocket, “veado gaedo”	Stroke	Terrestrial
*Mazama simplicicornis* (Illinger, 1811)	Diarrhoea, verminosis, evil eye	Terrestrial
*Mazama gouazoupira* (G. Fischer, 1814), grey brocket, “veado-catingueiro”	Asthma, edema, rheumatism, snake bite, thrombosis, assisting children who take longer than usual to start walking, toothache, wounds, sprains	Terrestrial
*Ozotocerus bezoarticus* (Linnaeus, 1758), Pampas Deer, veado campineiro	Diarrhoea, verminosis, evil eye	Terrestrial
Dasypodidae		
*Dasypus novemcinctus *(Linnaeus, 1758), nine-banded armadillo, “tatu galinha”	Thrombosis, insects bite, scorpions bite, edema, asthma, deafness, earache, evil eye	Terrestrial
*Euphractus sexcinctus *(Linnaeus, 1758), six-banded armadillo “tatu peba”	Wounds, earache, evil eye, asthma, sore throat, pneumonia, sinusitis, deafness, coarse throat	Terrestrial
*Tolypeutes tricinctus* (Linnaeus, 1758), Brazilian three-banded armadillo, “tatu-bola”	Thrombosis, rheumatism	Terrestrial
*Priodontes maximus *(Kerr, 1792), giant armadillo, tatu-canastra	Snake bite	Terrestrial
Dasyproctidae		
*Dasyprocta prymnolopha* Wagler, 1831, black-rumped agouti, “Cutia”	Asthma, thrombosis, earache	Terrestrial
Delphinidae		
*Sotalia fluviatilis *Gervais & Deville (1853), grey dolphin, grey river dolphin, “boto”	Asthma, headache, rheumatism, hernia, womb disorders, sore throat, injuries caused by the spines of the “arraia,” swelling, haemorrhoids inflammation, wounds, earache, erysipelas, athlete's foot, tumour, cancer	Freshwater
*Sotaliaguianensis* (P. J. Van Bénéden, 1864), Guianan River Dolphin, “boto”	Asthma, headache, rheumatism, hernia, womb disorders, sore throat, injuries caused by the spines of the “arraia,” swelling, haemorrhoids inflammation, wounds, earache, erysipelas, athlete's foot, tumour, cancer	Marine
Didelphidae		
*Didelphis albiventris *(Lund, 1840), White-Eared Opossum, “timbú”	Boils	Terrestrial
*Didelphis marsupialis *(Linnaeus, 1758), Black-Eared Opossum, “mucura,” “gambá,” “saruê”	Acne, wounds, bronchitis, joint pain, stomach ache, rheumatism, diarrhoea, inflammation, erysipelas, pain in gestation, asthma, headache, toothache, earache, sore throat	Terrestrial
Echimyidae		
*Thrichomys laurentius *Thomas, 1904, “punaré”	Diarrhoea	Terrestrial
Erethizontidae		
*Coendou prehensilis *(Linnaeus, 1758), Brazilian porcupine, “coandú,” “porco espinho”	Bronchitis, thrombosis, epilepsy, stroke, abscesses, conjunctivitis, asthma	Terrestrial
Hydrochaeridae		
*Hydrochaeris hydrochaeris *(Linnaeus, 1766), capybara, “capibara,” “capivara”	Thrombosis, conjunctivitis, venereal disease, rheumatism, earache, strengthen bones, liver pain, bronchitis, asthma, wounds, erysipelas, cough	Terrestrial
Iniidae		
*Inia geoffrensis *(Blainville, 1817), Amazon river dolphin, “boto rosa”	Asthma, headache, rheumatism, hernia, womb disorders, sore throat, injuries caused by the spines of the “arraia,” swelling, haemorrhoids inflammation, wounds, earache, erysipelas, athlete's foot, tumour, cancer	Freshwater
Leporidae		
*Sylvilagus brasiliensis *(Linnaeus, 1758), forest rabbit, tapeti, “coelho,” “coelho-do-mato”	Thrombosis, conjunctivitis, boils, burns	Terrestrial
Mustelidae		
*Conepatus semistriatus* (Boddaert, 1785), striped hog-nosed skunk, “cangambá,” “gambambá,” tacaca	Rheumatism	Terrestrial
*Lontra longicaudis* (Olfers, 1818), Neotropical Otter, “Lontra”	Thrombosis	Terrestrial
Myrmecophagidae		
*Myrmecophaga tridactyla* Linnaeus, 1758, giant anteater, “tamanduá-bandeira”	Thrombosis, stroke	Terrestrial
*Myrmecophaga tetradactyla *(Linnaeus, 1758), collared anteater, “tamanduá”	Edema, thrombosis	Terrestrial
Procyonidae		
*Nasua nasua *(Linnaeus, 1766), South American coati, “coati,” “quati”	Sexual impotence, wounds, skin burns, snake bites, backache	Terrestrial
*Procyon cancrivorus *(G. [Baron] Cuvier, 1798), crab-eating raccoon, “guaxinim”	Rheumatism, epilepsy, thrombosis, snake bite	Terrestrial
Tapiridae		
*Tapirus terrestris *(Linnaeus, 1758), Brazilian tapir, “anta”	Rheumatism, arthrosis, osteoporosis, bursitis, muscular pain, asthma, tonsillitis	Terrestrial
Tayassuidae		
*Pecari tajacu* Linnaeus 1758, collared peccary, “porco-do-mato,” “caititu”	Thrombosis, bronchitis, stroke	Terrestrial
*Tayassu pecari* (Link, 1795), white-lipped peccary “porco-do-mato,” “queixada”	Thrombosis, stroke	Terrestrial
Trichechidae		
*Trichechus inunguis *(Natterer, 1883), Amazonian manatee, “peixe-boi”	Sprains, vaginal discharge, injuries caused by bang, burns, asthma, menstrual cramps, rheumatism, sore throat, wounds, muscle strain, sucking a splinter out of skin or flesh, tumour, backache, hernia, arthrosis, luxation, menstrual cramps, insects bite	Freshwater
*Trichechus manatus *(Linnaeus, 1758), West Indian Manatee, “peixe-boi”	Sprains, vaginal discharge, injuries caused by bang, burns, asthma, menstrual cramps, rheumatism, sore throat, wounds, muscle strain, sucking a splinter out of skin or flesh, tumour, backache hernia, arthrosis, luxation, menstrual cramps, insects bite	Marine
Felidae		
*Puma concolor* (Linnaeus, 1771), mountain lion, “onça”	Wounds, leishmaniosis	Terrestrial
*Panthera onca* (Linnaeus, 1758), jaguar, “onça”	Wounds, leishmaniosis	Terrestrial
*Herpailurus yagouaroundi *(É. Geoffroy Saint-Hilaire, 1803), “gatovermelho,” “gato-azul,” Jaguarundi	Wounds	Terrestrial
*Leopardus pardalis* (Linnaeus, 1758), ocelot, “gato-maracajá,”	Headache, sore throat, backache, wounds	Terrestrial
*Leopardus tigrinus* (Schreber, 1775), oncilla, “gato-mirim”	Wounds, urinary incontinence, injuries, sore throat, sucking a splinter out of skin or flesh	Terrestrial

**Table 2:** Medicinal uses of edible fishes and game species in Brazil. Health condition categories follow the International Statistical Classification of Diseases and Related Health Problems (ICD-10 Version: 2016). *N* = total number of conditions treated in each category.

ICD 10	Indication of use and therapeutic properties	*N*
Symptoms, signs, and abnormal clinical and laboratory findings, not elsewhere classified (*n* = 58 species)	Ascites; chest pain; cough; cracks in the sole of the feet; edema (also quoted as edema in the legs); fatigue; fever; headache; hoarseness; inflammation; jaundice; lack of appetite (also quoted as lack of appetite in children); numbness; pain (also quoted as pain in the body; pain in the breast; pain in the legs; to reduce pain); shortness of breath; swelling; assisting children who take longer than usual to start walking; vomit.	24
Certain infectious and parasitic diseases (*n* = 40 species)	Abscesses; athlete's foot; diphtheria; erysipelas; herpes zoster; infection; leishmaniosis; leprosy; mumps; mycosis; schistosomiasis; syphilis; tetanus; tuberculosis; venereal disease; verminosis; warts; whooping cough.	18
Injury, poisoning, and certain other consequences of external causes (*n* = 67 species)	Bruises; burns (also quoted as burns in the skin); chilblains; injuries caused by bang; injuries caused by the animal itself; injuries caused by the spines of fishes (also quoted as injuries caused by the spines of rays); intoxication from poisonous animals; pain relief in injuries caused by the species' sting; pain relief in injuries caused by snake bites; pain relief in injuries caused by sting of insects; scratch; assisting in removing spines or other sharp structures from the skin (also quoted as to suck a splinter out of skin or flesh); wounds.	16
Diseases of the digestive system (*n* = 40 species)	Appendicitis; constipation; dental caries; diarrhoea; gastritis; haemorrhoids; hernia (also quoted as umbilical hernia); indigestion; irritation when milk teeth are erupting; liver pain; stomach ache; stomach disorders; toothache.	14
Diseases of the musculoskeletal system and connective tissue (*n* = 80 species)	Arthritis; arthrosis; backache; bursitis; luxation; muscle strain; muscular pain; neck strain; osteoporosis; pain in joint; rheumatism; sprains; helping to strengthen bones.	13
Diseases of the respiratory system (*n* = 80 species)	Asthma; bronchitis; catarrh; coarse throat; cold; flu; lung disease; pneumonia; respiratory diseases; sinusitis; sore throat; tonsillitis.	12
Diseases of the skin and subcutaneous tissue (*n* = 21 species)	Acne; blackhead; boils; calluses; dermatitis; itching; paronychia; skin diseases; skin spots; skin thorns and wounds; wrinkles.	11
Diseases of the genitourinary system (*n* = 19 species)	Menstrual cramps; nephritis; prostate problems; renal failure; urinary disorders; urinary incontinence; urinary infection; discharge (also quoted as vaginal discharge); womb disorders.	10
Pregnancy, childbirth, and the puerperium (*n* = 12 species)	Disorders after parturition (to accelerate recovery after parturition); haemorrhage after delivery; nausea during pregnancy; pain in gestation; helping to accelerate parturition; helping to avoid swelling of the breast feeding; helping to induce abortion; helping to prevent abortion; wound in the breast caused by suckling.	9
Diseases of the eye and adnexa (*n* = 13 species)	Cataracts; conjunctivitis; eye pains; ophthalmological problems (also quoted as eye problems); leucoma; pterygium; stye.	8
External causes of morbidity and mortality (*n* = 25 species)	Dog bite; insect sting; scorpions sting; snake bite; helping to stop the sensation of getting thirsty.	5
Undefined (*n* = 11 species)	Amulet; amulet used as a protection against snake bite; evil eye; helping to remove wrath.	4
Diseases of the blood and blood-forming organs and certain disorders involving the immune mechanism (*n* = 12 species)	Anaemia; bleeding (also quoted as wounds bleeding); haemorrhage.	4
Diseases of the circulatory system (*n* = 42 species)	Stroke; thrombosis; varicocele.	3
Diseases of the nervous system (*n* = 5 species)	Epilepsy; insomnia; paralysis of arms and legs.	3
Mental and behavioural disorders (*n* = 13 species)	Alcoholism; sexual impotence; helping to stay awake.	3
Endocrine, nutritional, and metabolic diseases (*n* = 7 species)	Diabetes, goitre; helping to control cholesterol level.	3
Neoplasms (*n* = 18 species)	Breast cancer; cancer (also quoted as tumour).	3
Diseases of the ear and mastoid process (*n* = 40 species)	Deafness; earache.	2
Certain conditions originating in the perinatal period (*n* = 1 species)	Healing of umbilical cord of newborn baby.	1
